# Hospital Readmission After Traumatic Brain Injury Hospitalization in Community‐Dwelling Older Adults

**DOI:** 10.1002/acn3.70269

**Published:** 2025-11-30

**Authors:** Rachel Thomas, Connor A. Law, Joan A. Casey, Thomas Mosley, Rebecca F. Gottesman, Ramon Diaz‐Arrastia, Holly Elser, Andrea L. C. Schneider

**Affiliations:** ^1^ Department of Neurology University of Pennsylvania Perelman School of Medicine Philadelphia Pennsylvania USA; ^2^ Department of Environmental and Occupational Health Sciences University of Washington School of Public Health Seattle Washington USA; ^3^ Department of Epidemiology University of Washington School of Public Health Seattle Washington USA; ^4^ The MIND Center University of Mississippi Medical Center Jackson Mississippi USA; ^5^ National Institute of Neurological Disorders and Stroke Intramural Research Program National Institutes of Health Bethesda Maryland USA; ^6^ Department of Biostatistics, Epidemiology, and Informatics University of Pennsylvania Perelman School of Medicine Philadelphia Pennsylvania USA

**Keywords:** hospital readmissions, TBI, traumatic brain injury

## Abstract

**Objective:**

To examine the risk of hospital readmission after an index hospitalization for TBI in older adults.

**Methods:**

Using data from the Atherosclerosis Risk in Communities (ARIC) study, we used propensity score matching of individuals with an index TBI‐related hospitalization to individuals with (1) non‐TBI hospitalizations (primary analysis) and (2) orthopedic injury hospitalizations (secondary analysis). The rate of all‐cause hospital readmission was estimated using adjusted Fine‐Gray proportional hazards regression models within strata defined by time since hospitalization. Follow‐up extended from the date of index hospitalization after study enrollment (1987–1989) through December 31, 2019.

**Results:**

Six hundred sixty‐seven participants with an index TBI‐related hospitalization were matched using propensity scores with replacement with a ratio of 1:4–2668 participants with non‐TBI‐related hospitalizations and 1:3 with 2001 participants with orthopedic injury‐related hospitalizations. Median age was 68 years (IQR = 46–95) at index hospitalization. Compared to participants with an index non‐TBI‐related hospitalization, readmission rates were lower among participants with an index TBI‐related hospitalization (HR = 0.80, 95% CI = 0.74, 0.87) over a median follow‐up of 2.7 years, although similar readmission rates were observed within the first year of follow‐up (HR = 1.03, 95% CI = 0.88, 1.21). Rates of hospital readmission among persons with an index TBI‐related hospitalization were similarly decreased compared with persons with an orthopedic injury‐related hospitalization (HR = 0.78, 95% CI = 0.72, 0.85) over a median follow‐up of 3.1 years.

**Interpretation:**

Among community‐dwelling older adults with a TBI‐related hospitalization, rates of hospital readmission were similar to individuals hospitalized for a non‐TBI cause in the first year, but lower subsequently. Targeted interventions in the first year post‐hospitalization may be most beneficial for reducing readmissions among individuals with TBI.

## 1 Introduction

The annual incidence of traumatic brain injury (TBI) approaches 69 million individuals worldwide [1]. In the United States (US) alone, approximately 2.8 million individuals seek medical care for TBI in emergency departments, resulting in over 282,000 hospitalizations and 56,000 deaths each year [2, 3, 4, 5]. On average, initial TBI hospitalizations are longer in duration, costlier, and carry a higher mortality rate than non‐TBI admissions [3]. Older adults have the highest rates of TBI, with individuals aged ≥ 75 years accounting for 32% of all TBI‐related hospitalizations [4].

In prior studies that have leveraged administrative data, all‐cause hospital readmissions are common among individuals with prior TBI, approaching 14% within one year [6] and 20%–23% within the first three years after the index injury [7]. Prior studies have identified several risk factors for hospital readmission after TBI in the acute post‐injury period, including male sex, older age, history of falls, and public insurance [6, 8, 9, 10]. Clinical factors associated with increased risk for hospital readmission include greater injury severity, intracerebral hemorrhage [3], diagnosis of acute‐symptomatic seizures during index admission [11], and discharge to a skilled nursing facility or against medical advice [9]. However, prior research is generally descriptive, limited to individuals with TBI with no comparison population, or focuses on readmission risk among younger populations (i.e., mean age ≤ 50 years) [3, 6, 7, 8, 9, 10]. This leaves important knowledge gaps regarding the risk of rehospitalization after TBI hospitalization among community‐dwelling older adults.

Building on prior research, the present study uses prospectively collected data from the Atherosclerosis Risk in Communities (ARIC) Study to examine the rates of hospital readmission following an index TBI hospitalization in community‐dwelling older adults. Our analysis compares persons with TBI‐related hospitalization to two distinct matched comparison cohorts: (1) persons with non‐TBI hospitalizations and (2) persons with orthopedic injury‐related index hospitalizations. To characterize rates of hospital readmission over time, we conducted analyses stratified by time since index hospitalization.

## 2 Methods

### 2.1 Study Population

The ARIC Study is an ongoing prospective cohort study comprised of community‐dwelling adults who were aged 45–64 years at study baseline in 1987–1989. Participants were recruited from four US communities in Forsyth County, North Carolina; Jackson, Mississippi; Washington, County, Maryland; and the northwest suburbs of Minneapolis, Minnesota. The Minneapolis, Minnesota and Washington County, Maryland centers recruited mainly White participants, and the Forsyth County, North Carolina center recruited mainly White and Black participants (based on probability sampling of these communities), while the Jackson, Mississippi center recruited only Black participants. Participants have been followed through in‐person visits as well as annual (1988–2011) and later semi‐annual (2012 onward) telephone interviews [12]. The ARIC study collects hospitalization data through active surveillance of all hospitalizations occurring in the study community hospitals and via annual/semi‐annual telephone contact with study participants. If a hospitalization occurring outside of study communities is reported during telephone contact, records are subsequently sought. Follow‐up for the present study extended from the date of index hospitalization until first rehospitalization, loss to follow‐up, withdrawal from the study, death, or administrative censoring on December 31, 2019.

The ARIC Study is approved by the Institutional Review Boards of all participating institutions, and all participants (or legally authorized representatives) provided written informed consent to participate in each study visit. This study is reported in concordance with the Strengthening of Reporting in Observational Epidemiology (STROBE) guidelines.

### 2.2 Index TBI Hospitalizations

Index TBI events requiring hospital care were identified from the ARIC Study hospitalization data using discharge diagnostic codes from the *International Classification of Diseases, Ninth* (ICD‐9) and *Tenth Revisions* (ICD‐10) according to the Centers for Disease Control and Prevention (CDC) surveillance case definition for TBI (Table S1) [13, 14, 15, 16]. Only the first TBI‐related hospitalization for each individual was considered in analyses. As TBI often co‐occurs with other bodily trauma, we considered hospitalizations with an ICD code for TBI in any diagnostic position as TBI‐related hospitalizations.

### 2.3 Index Non‐TBI and Orthopedic Injury‐Related Hospitalizations

We considered two comparison groups: (1) non‐TBI hospitalizations (primary analysis) and (2) orthopedic injury hospitalizations (secondary analysis). Non‐TBI hospitalizations were defined as any hospitalizations occurring without associated discharge ICD‐9/10 codes for TBI occurring among participants who never had a hospitalization for TBI. Orthopedic injury‐related hospitalizations were defined as hospitalizations with ICD‐9/10 codes for fractures and/or falls (Table S2) without associated ICD‐9/10 codes for TBI occurring among participants who never had a hospitalization for TBI.

### 2.4 Hospital Readmissions

The main outcome of interest was incident all‐cause hospital readmission (i.e., an admission after the index hospitalization) identified through the ARIC hospitalization data. We also examined cause‐specific readmissions, classified based on the ICD‐9 and ICD‐10 diagnostic codes in the first position into the following categories: cardiovascular; pulmonary; allergic/immunologic and rheumatologic; dermatologic; orthopedic; muscular; endocrine, nutritional or toxic/metabolic; gastrointestinal; renal and genitourinary; gynecologic; infectious; oncologic; hematologic; neurologic; ophthalmologic; otolaryngologic; psychiatric; and other [17].

### 2.5 Variables Included in Models to Create Propensity Scores

Variables included in the models to create the propensity scores are those that are conceptualized as confounders of the association of TBI with hospital readmission risk; these variables were selected a priori, based on the prior literature [18, 19, 20]. Variables defined at baseline (Visit 1) included sex (male; female), race/center (due to race‐center aliasing, categorized as Maryland White; Minnesota White; North Carolina White; North Carolina Black; Mississippi Black), education (less than high school, high school or equivalent, some college or greater), annual household income (greater than or equal to median of $35,000; below median of $35,000; not reported), health insurance status (insured; uninsured), military veteran status (yes, no), and an index of physical activity ascertained from the modified Baecke Physical Activity Questionnaire [21]. Variables defined at the closest visit prior to the index hospitalization were defined in a consistent manner over calendar time and included body mass index (kg/m^2^; continuous), smoking (current, former, never), alcohol consumption (current, former, never), hypertension (yes; no, defined as systolic blood pressure ≥ 140 mmHg, diastolic blood pressure ≥ 90 mmHg, or use of anti‐hypertensive medication), hyperlipidemia (yes; no, defined as total cholesterol ≥ 200 or use of hyperlipidemia medications), diabetes (yes; no, defined as fasting glucose ≥ 126 mg/dL, non‐fasting glucose ≥ 200 mg/dL, self‐reported physician diagnosis, or use of medication for diabetes), coronary heart disease (yes; no, self‐reported at Visit 1 with ongoing adjudicated events defined as myocardial infarction, heart or arterial surgery, coronary artery bypass graft surgery, or angioplasty; or evidence of prior myocardial infarction based on electrocardiogram) [22], and stroke (yes; no, self‐reported at Visit 1, ongoing physician adjudication of definite/probable ischemic or hemorrhagic events) [23]. Age on the date of the index hospitalization event was also included (years; continuous).

### 2.6 Statistical Analyses

To minimize confounding bias, we used propensity scores to match individuals with incident index TBI hospitalization to individuals with non‐TBI‐related hospitalizations who were otherwise comparable using full matching with replacement [24, 25]. For individuals without TBI‐related hospitalizations, we considered all hospitalizations in the pool of potential matches. Propensity scores were estimated using the following covariates (as defined above): age, sex, race/center, education, income, insurance, marital status, military veteran status, physical activity, body mass index, smoking, alcohol consumption, hypertension, hyperlipidemia, diabetes, coronary heart disease, stroke, and date of the index TBI hospitalization [26]. Given that TBI is often associated with systemic forms of trauma (i.e., polytrauma) that may confound our results, as a secondary analysis we also compared participants with incident index TBI hospitalizations to individuals hospitalized for orthopedic injuries (fractures and/or falls). This study design is consistent with that of several large TBI cohorts, including the Transforming Research and Clinical Knowledge in TBI (TRACK‐TBI) Study [27].

Propensity scores were estimated using SuperLearner [28], an ensemble machine‐learning approach that has been previously shown to improve propensity score estimation [29, 30]. SuperLearner optimally weights multiple machine learning algorithms (we used generalized linear models [GLM], Bayesian GLM, multivariate adaptive regression splines), and also included the mean of the probability of a TBI hospitalization as a benchmark algorithm to minimize 10‐fold cross‐validated prediction error. The advantage of this approach is reduced reliance on the correct specification of the parametric exposure model for propensity score estimation. We placed the following restrictions on matching: age was required to be within 3 years, index hospitalization date was required to be within 3 years, and sex was required to be an exact match. To reduce model extrapolation, we windsorized the propensity scores of hospitalizations with a score below the 1st propensity score percentile and above the 99th propensity score percentile [31]. Due to the number of available eligible hospitalizations, participants with TBI‐related hospitalizations were matched 1:4 with non‐TBI hospitalizations for our primary analysis and 1:3 with orthopedic injury‐related hospitalizations for our secondary analysis. Quality of the match was evaluated based on the standardized mean differences between matching variables and via visual inspection of the distribution of propensity scores in the matched groups [20, 26].

Within our matched analytic samples, Kaplan–Meier analyses were used to estimate the probability of rehospitalization‐free survival for each group. We used Fine‐Gray proportional hazards regression models to examine the instantaneous rate (subdistribution hazard) of all‐cause incident hospital re‐admission, accounting for the competing risk of death. The proportional subdistribution hazard assumption was confirmed using the goodness‐of‐fit test for proportional subdistribution hazards models described by Zhou, Fine, and Laird [32] (based on modified weighted Schoenfeld residuals). Our analysis considered the overall risk of hospital readmissions and further characterized the risk of hospital readmission based on time elapsed since the index hospitalization date (< 1 month; 1 month to 1 year; 1 year to 5 years; ≥ 5 years).

All statistical analyses were performed with R version 4.4.2 (R Foundation for Statistical Computing, Vienna, Austria). A two‐sided *p* < 0.05 was considered statistically significant.

## 3 Results

### 3.1 Participant Characteristics

Of the 15,792 ARIC participants initially enrolled, we excluded 34 Asian, 14 Native American, and 55 Black individuals at the Maryland and Minnesota field centers (due to small numbers and race‐center aliasing). We additionally excluded 1151 individuals with no hospitalizations recorded over the study period, and 3 with missing hospitalization dates, as well as 2136 with hospitalizations occurring only after the administrative censoring date of December 31, 2019, resulting in 12,399 participants with eligible hospitalizations. Among these 12,399 participants, after excluding those without a full complement of covariates (*n* = 129 participants), there were 667 incident index TBI‐related hospitalizations, 44,229 eligible non‐TBI hospitalizations, and 5379 eligible orthopedic injury‐related hospitalizations.

**FIGURE 1:**
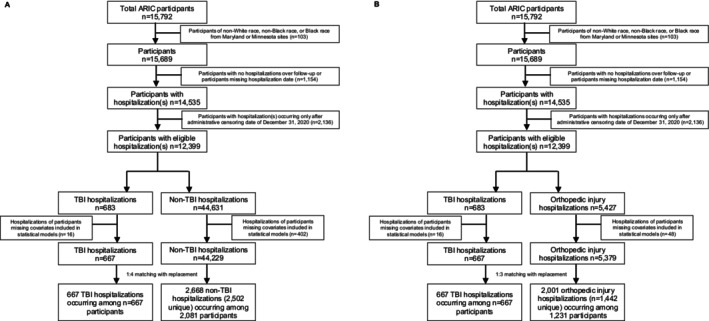
Participant and hospitalization inclusion and exclusions for analyses. Panel A: TBI versus non‐TBI index hospitalization analysis and Panel B: TBI versus orthopedic injury index hospitalization analysis.

After propensity matching, the final analytic samples included: (1) 667 TBI‐related hospitalizations occurring among 667 participants matched with replacement to 2668 non‐TBI‐related hospitalizations (2502 of which were unique) occurring among 2081 unique participants (Figure 1A); and (2) 667 TBI‐related hospitalizations occurring among 667 participants matched with replacement to 2001 orthopedic injury‐related hospitalizations (1442 of which were unique) occurring among 1231 unique participants (Figure 1B). Among the 667 participants with an index TBI‐related hospitalization, 35% also had associated orthopedic injury.

In the primary analysis sample of participants with TBI‐related index hospitalizations and with non‐TBI‐related index hospitalizations, participants were of median age 68 years (IQR: 46–95) at the time of index hospitalization (Table 1). The majority (81.4%) were female, and 22.6% self‐identified as Black race. Similarly, in the secondary analysis sample of participants with TBI‐related index hospitalizations and with orthopedic injury‐related index hospitalizations, participants were of median age 68 years (IQR: 46–95) at index hospitalization, 81.4% were female and 22.6% self‐reported Black race. Comparing participants with TBI versus non‐TBI‐related index hospitalizations and comparing participants with TBI versus orthopedic injury‐related index hospitalizations (Table S3), standardized mean differences for all characteristics included in the propensity score model were < 0.1, which suggests propensity score matching achieved satisfactory balance of covariates across groups.

**TABLE 1:** Participant characteristics.^a^

	Index TBI hospitalization (*n* = 667 hospitalizations among *n* = 667 participants)^a^	Index non‐TBI hospitalization (*n* = 2668 hospitalizations; *n* = 2502 unique hospitalizations among *n* = 2081 participants)^a^	Index orthopedic injury hospitalization (*n* = 2001 hospitalizations; *n* = 1442 unique hospitalizations among *n* = 1231 participants)^a^
Age at index hospitalization (years), median (IQR)	68 (46, 95)	67 (46, 95)	68 (46, 95)
Sex, *n* (%)			
Female	543 (81.4)	2172 (81.4)	1629 (81.4)
Male	124 (18.6)	496 (18.6)	372 (18.6)
Race/center, *n* (%)			
Mississippi Black	135 (20.2)	514 (19.3)	390 (19.5)
North Carolina Black	20 (3.0)	86 (3.2)	57 (2.8)
Maryland White	165 (24.7)	645 (24.2)	520 (26.0)
Minnesota White	202 (30.3)	808 (30.3)	601 (30.0)
North Carolina White	145 (21.7)	615 (23.1)	433 (21.6)
Education, *n* (%)			
Less than high school	133 (19.9)	483 (18.1)	394 (19.7)
High school or equivalent	272 (40.8)	1146 (43.0)	819 (40.9)
Some college or greater	262 (39.3)	1039 (38.9)	788 (39.4)
Annual household income, *n* (%)			
< $35,000	362 (54.3)	1407 (52.7)	1088 (54.4)
≥ $35,000	272 (40.8)	1146 (43.0)	802 (40.1)
Not reported	33 (4.9)	115 (4.3)	111 (5.5)
Health insurance, *n* (%)	604 (90.6)	2437 (91.3)	1838 (91.9)
Military veteran, *n* (%)	81 (12.1)	332 (12.4)	251 (12.5)
Physical activity index, median (IQR)	2.25 (1.00, 5.00)	2.25 (1.00, 5.00)	2.25 (1.00, 5.00)
Body mass index (kg/m^2^), median (IQR)	28.1 (16.2, 55.0)	28.0 (14.4, 60.2)	28.0 (15.3, 54.1)
Smoking, *n* (%)			
Current	120 (18.0)	497 (18.6)	360 (18.0)
Former	243 (36.4)	945 (35.4)	716 (35.8)
Never	304 (45.6)	1226 (46)	925 (29.1)
Alcohol consumption, *n* (%)			
Current	322 (48.3)	1293 (48.5)	951 (47.5)
Former	163 (24.4)	655 (24.6)	467 (23.3)
Never	182 (27.3)	720 (27.0)	583 (29.1)
Hypertension, *n* (%)	337 (50.5)	1364 (51.1)	992 (49.6)
Hyperlipidemia, *n* (%)	425 (63.7)	1734 (65.0)	1282 (64.1)
Diabetes, *n* (%)	125 (18.7)	504 (18.9)	367 (18.3)
Coronary artery disease, *n* (%)	50 (7.5)	212 (7.9)	139 (6.9)
Stroke, *n* (%)	14 (2.1)	57 (2.1)	44 (2.2)

*Note:* The following variables were ascertained at Visit 1 (1987–1989): sex, race/center, education, annual household income, health insurance status, military veteran status, physical activity index. The following variables were defined at the closest visit prior to the index hospitalization: body mass index, smoking, alcohol consumption, hypertension, hyperlipidemia, diabetes, coronary heart disease, stroke.

Abbreviations: IQR, interquartile range; TBI, traumatic brain injury.

^a^ Index TBI hospitalizations were matched 1:4 with replacement to index non‐TBI hospitalizations and 1:3 with replacement to index orthopedic injury hospitalizations; data shown in this table is representative of participant characteristics associated with each hospitalization.

### 3.2 TBI Versus Non‐TBI‐Related Index Hospitalization

**FIGURE 2:**
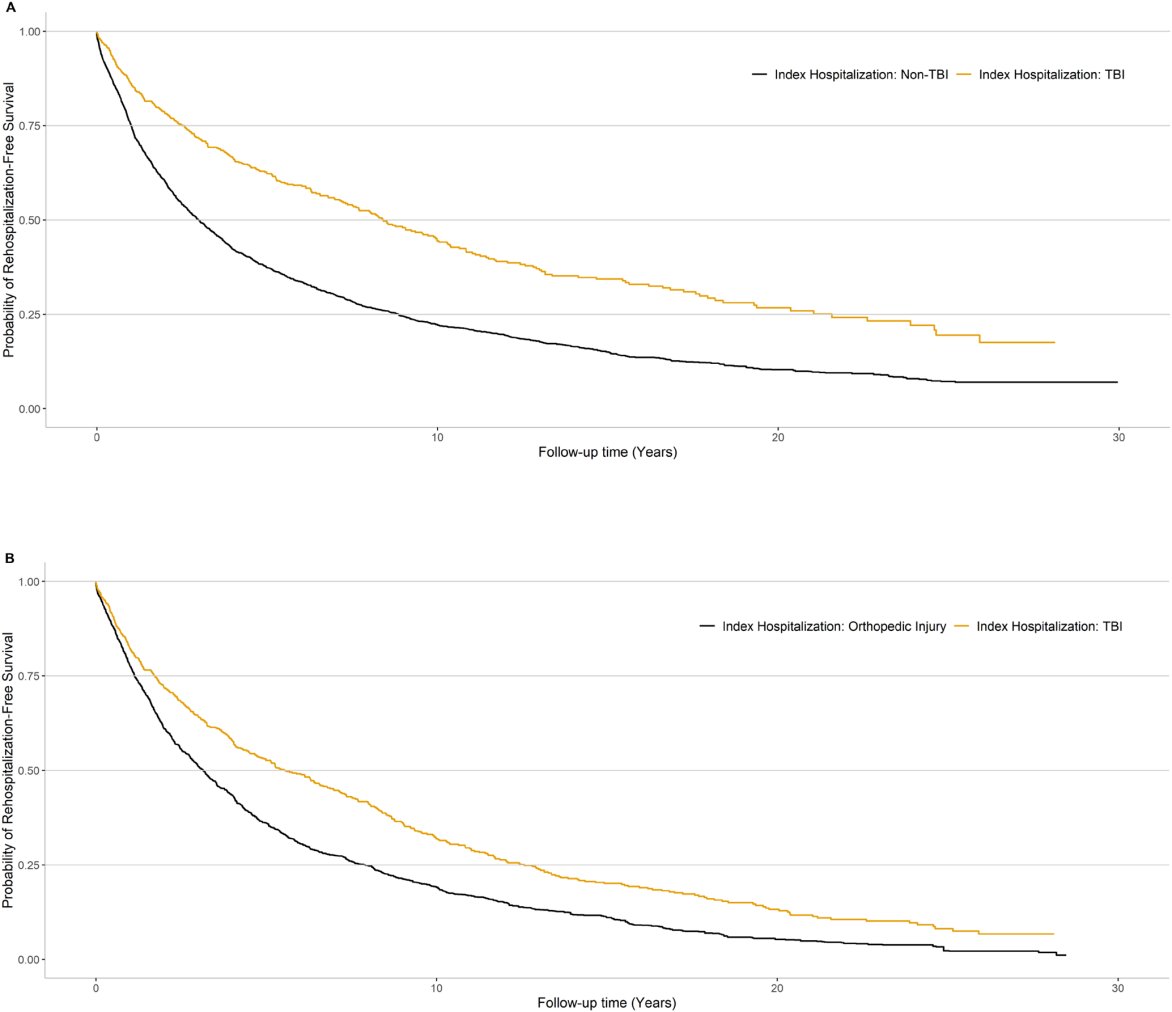
Probability of rehospitalization‐free survival by index hospitalization group. Panel A: TBI versus non‐TBI index hospitalization and Panel B: TBI versus orthopedic injury index hospitalization.

**FIGURE 3:**
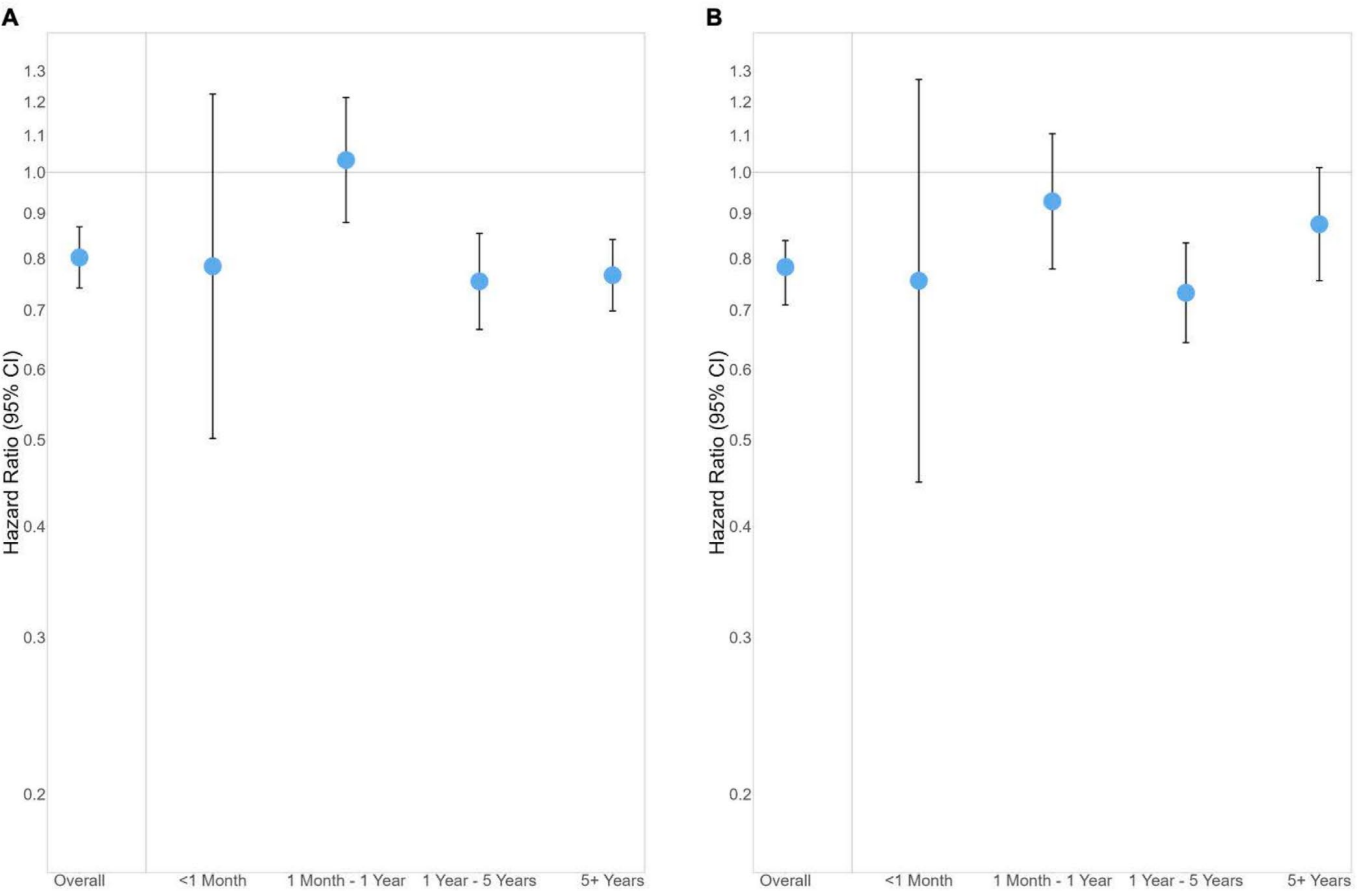
Risk of hospital readmission overall and stratified by follow‐up time from Fine‐Gray proportional hazards regression models. Panel A: TBI versus non‐TBI index hospitalization analysis and Panel B: TBI versus orthopedic injury index hospitalization analysis.

**FIGURE 4:**
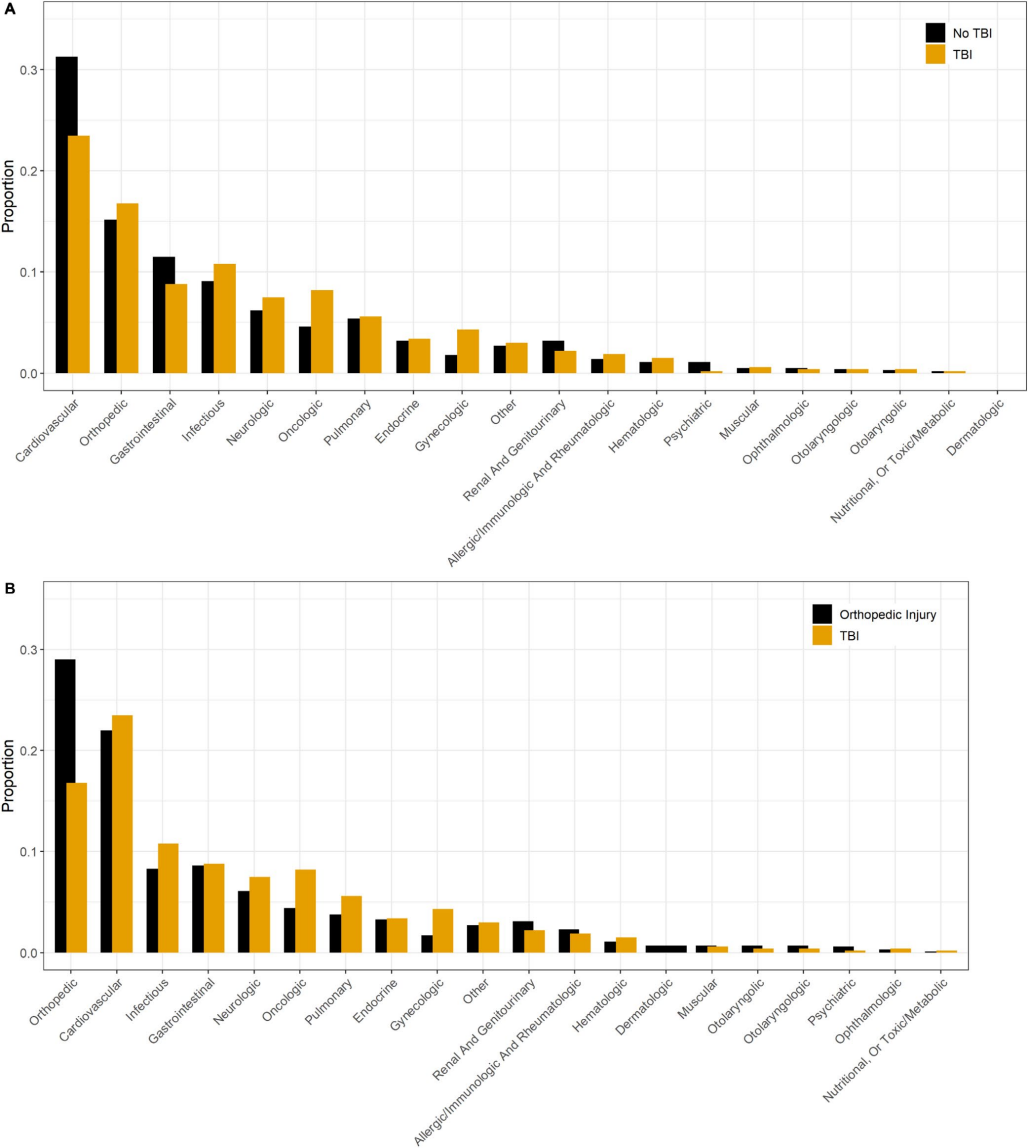
Primary causes of hospital readmissions defined based on the ICD‐9 and ICD‐10 diagnostic codes in the first position. Panel A: TBI versus non‐TBI index hospitalization analysis and Panel B: TBI versus orthopedic injury index hospitalization analysis.

In the primary analysis comparing participants with TBI versus non‐TBI‐related index hospitalizations, 2473 incident hospital readmissions occurred over a median follow‐up period of 2.7 years (IQR: 0.9–7.2). The cumulative hospital readmission‐free survival was consistently slightly higher for participants with an index TBI hospitalization compared to those with an index non‐TBI hospitalization (Figure 2A). Overall, compared to participants with an index non‐TBI‐related hospitalization, participants with an index TBI‐related hospitalization were less likely to have an incident hospital readmission (HR: 0.80, 95% CI: 0.74, 0.87) (Figure 3A, Table 2). In analyses stratified by follow‐up time, the risk of incident hospital readmission was similar between the TBI and non‐TBI index hospitalization groups over the first year post‐index hospitalization (HR: 1.03, 95% CI: 0.88, 1.21), although estimates for within 1 month were imprecise (HR: 0.78, 95% CI: 0.50, 1.23). Participants with index TBI‐related hospitalizations were less likely to have an incident hospital readmission after the first year post‐index hospitalization compared to participants with index non‐TBI‐related hospitalization (HR: 0.75, 95% CI: 0.67, 0.85) for 1–5 years post‐index hospitalization and HR: 0.77, 95% CI: 0.70, 0.84 for ≥ 5 years post‐index hospitalization. In analyses evaluating causes of readmission, cardiovascular and orthopedic causes were the first and second most common causes for readmission for participants with both index TBI and non‐TBI‐related hospitalization (Figure 4A), however, those with an index TBI were more likely to be readmitted for infectious, neurologic, and oncologic causes.

**TABLE 2:** Risk of hospital readmission overall and stratified by follow‐up time.

	Index non‐TBI hospitalization	Index TBI hospitalization	Index orthopedic injury hospitalization	Index TBI hospitalization
Overall				
# Rehospitalizations/PYs	2009/13,227	464/3997	1533/10,282	464/3997
HR (95% CI)	1 (Reference)	0.80 (0.74, 0.87)	1 (Reference)	0.78 (0.72, 0.85)
< 1 Month				
# Rehospitalizations/PYs	114/4.25	17/0.67	66/2.14	17/0.67
HR (95% CI)	1 (Reference)	0.78 (0.50, 1.23)	1 (Reference)	0.76 (0.45, 1.27)
1 Month to 1 year				
# Rehospitalizations/PYs	446/319	98/71	305/211	98/71
HR (95% CI)	1 (Reference)	1.03 (0.88, 1.21)	1 (Reference)	0.93 (0.78, 1.11)
1 Year to 5 years				
# Rehospitalizations/PYs	853/2788	168/642	684/2241	168/642
HR (95% CI)	1 (Reference)	0.75 (0.67, 0.85)	1 (Reference)	0.73 (0.64, 0.83)
≥ 5 Years				
# Rehospitalizations/PYs	1449/12,913	349/3928	478/7835	181/3286
HR (95% CI)	1 (Reference)	0.77 (0.70, 0.84)	1 (Reference)	0.87 (0.76, 1.01)

### 3.3 TBI Versus Orthopedic Injury‐Related Index Hospitalization

In the secondary analysis comparing participants with TBI versus orthopedic injury‐related index hospitalizations, 1997 incident hospital readmissions occurred over a median of 3.1 years (IQR: 1.1–7.7) of follow‐up. The cumulative hospital readmission‐free survival was consistently slightly higher for participants with an index TBI hospitalization compared to those with an index orthopedic injury‐related hospitalization (Figure 2B). Compared to participants with an index orthopedic injury‐related hospitalization, participants with an index TBI‐related hospitalization were less likely to have an incident hospital readmission overall (HR: 0.78, 95% CI: 0.72, 0.85) (Figure 3B, Table 2). Participants with index TBI‐related hospitalization had similar rates of rehospitalization within 1 year of the index hospitalization compared to persons hospitalized for orthopedic injury (HR: 0.93, 95% CI: 0.78, 1.11) and lower rates of readmission between 1 and 5 years post‐index hospitalization (HR: 0.73, 95% CI: 0.64, 0.83). The most common cause for readmission for participants with index orthopedic injury‐related hospitalizations was orthopedic causes, followed by cardiovascular, whereas this rank order of causes was reversed for those with an index TBI hospitalization (Figure 4B).

## 4 Discussion

Among community‐dwelling adults who sustain a TBI requiring hospitalization in older age, the risk of hospital readmission was similar to individuals hospitalized for non‐TBI causes within the first year, but lower in subsequent years. This pattern was largely consistent when comparing individuals hospitalized for TBI to individuals hospitalized for orthopedic injury without concomitant neurotrauma. Causes of hospital readmissions were primarily related to cardiovascular etiologies, though orthopedic causes were also common among those with TBI‐related hospitalization and in matched comparison groups.

Neurotrauma demographics have shifted over time, with the population impacted by TBIs becoming increasingly older [6]; prior studies have highlighted the impact of age on rehospitalizations [6, 8, 9, 10], with older adults having up to 40% higher readmission rates [6]. Prior work has examined risk factors associated with hospital readmissions among individuals hospitalized for TBI and has described a high proportion of adults with TBI re‐presenting to the hospital, especially within the first year following index hospitalization [3, 6, 7, 8, 9, 10]. This study extends upon this prior work by examining readmission risk relative to two separate comparison groups over a longer follow‐up. In keeping with prior studies, despite an overall lower relative readmission risk for participants with an index TBI‐related hospitalization compared to participants with an index non‐TBI or orthopedic trauma‐related hospitalization, the absolute incidence of hospital readmission was still quite high among participants with an index TBI‐related hospitalization. In analyses stratified by follow‐up time, the risk of incident hospital readmission was similarly high between the TBI and non‐TBI index hospitalization groups over the first year post‐index hospitalization. This suggests a potential role for targeted interventions prior to discharge to mitigate short‐term rehospitalization risk among individuals with TBI‐related hospitalizations. In contrast, beyond the first year index hospitalization, readmission risk was significantly lower for the TBI group compared to the non‐TBI group. It is possible that individuals with TBI establish care with long‐term caregivers and support services, potentially leading to more careful observation and attention to health maintenance over time. Indeed, prior work has suggested that the use of home health services post‐TBI has increased over the past decade [6]. It is also possible that the non‐TBI comparison group may have had worse baseline health status compared to our TBI group. This might result in individuals with TBI having lower readmission risk once the acute injury‐related sequelae are resolved; however we included an orthopedic injury comparison group as a way to account for this possibility. Further, we reduced the risk of confounding bias by leveraging the detailed phenotype data in the ARIC Study to create propensity‐matched cohorts; however, the possibility of residual confounding by factors not included in our models remains. Our analyses also accounted for the competing risk of mortality, making differential mortality unlikely to account for the lower readmission rates after the first year post‐index hospitalization.

In both our primary and secondary analyses, the most common causes for readmission across all groups were cardiovascular and orthopedic in nature. Prior studies have suggested that individuals with TBI are at increased risk for the development of cardiovascular disease, stroke, and vascular risk factors, including hypertension, hyperlipidemia, and obesity [19, 33, 34]. Our results further emphasize the potential benefit of aggressive treatment of cardiovascular disease risk factors post‐injury. Readmissions for orthopedic injuries were quite common; however, we could not determine whether these readmissions represented complications of the index hospitalization event, de novo pathology, or both. However, prior work does suggest that individuals with TBI have an increased risk of subsequent falls [35]. The high number of readmissions for orthopedic etiologies suggests that further work examining the potential impact of targeted interventions, such as physical therapy targeted to improve gait and balance, is warranted.

Despite the methodological strengths of this study, it is subject to limitations that should be recognized. First, it is important to consider generalizability. The majority of this cohort is female, in contrast to the majority of TBIs occurring in the United States, which are recognized to primarily occur among males [36]. However, as has been previously reported [6], there has been a rise over the past few decades in the proportion of neurotrauma occurring among females. Indeed, epidemiological data suggest that the sex differences in TBI no longer exist beyond age 75 years [36, 37]. Even so, our analytic sample is > 80% female. It is possible that there are sex differences in the likelihood of being hospitalized for a TBI in older age, which may have contributed to the female majority in our study. Our study investigated the risk of readmissions occurring after TBI‐related index hospitalizations that were defined using the validated CDC surveillance case definition for TBI [38]. This minimizes misclassification of hospitalizations, but the possibility for some misclassification by TBI status remains as not all TBIs result in hospitalization and some individuals in the non‐TBI group may have suffered a TBI but not sought medical care or sought care only in the outpatient or urgent/emergency care setting.

## 5 Conclusion

In this study we found that TBI hospitalization in older community‐dwelling adults carries lower risk of readmission compared to non‐TBI as well as orthopedic injury‐related hospitalizations. However, this overall pattern is driven by the lower risk observed during multi‐year follow‐up; the risk of readmission was similar in the first year post‐index hospitalizations. Further work is needed to elucidate underlying contributors to the risk of readmissions after TBI, but this work suggests that causes of readmission are similar regardless of the cause of initial hospitalization, suggesting that prevention strategies focused on reduction of cardiovascular risk and orthopedic injury may be beneficial.

## Author Contributions

R.T., C.A.L., H.E., A.L.C.S. contributed to the conception and design of the study; R.T., C.A.L., J.A.C., H.E., A.L.C.S. contributed to the acquisition and analysis of data; R.T., C.A.L., J.A.C., T.M., R.F.G., R.D.‐A., H.E., A.L.C.S. contributed to drafting the text or preparing the figures.

## Funding

The Atherosclerosis Risk in Communities Study is carried out as a collaborative study supported by NIH/NHLBI contracts (75N92022D00001, 75N92022D00002, 75N92022D00003, 75N92022D00004, 75N92022D00005). The ARIC Neurocognitive Study is supported by U01HL096812, U01HL096814, U01HL096899, U01HL096902, and U01HL096917 from the NIH (NHLBI, NINDS, NIA and NIDCD). R.F.G. was supported by the NINDS Intramural Research Program. A.L.C.S. was supported by K23NS123340, DoD W81XWH‐21‐1‐0590, and DoD HT9425‐23‐1‐0981.

## Conflicts of Interest

The authors declare no conflicts of interest.

## Supporting information


**Table S1:** Table of discharge ICD‐9/10 codes for traumatic brain injury (TBI).
**Table S2:** Table of discharge ICD‐9/10 codes for orthopedic injuries (including falls and fractures).
**Table S3:** Standardized mean differences in variables comparing pre‐ versus post‐propensity score matching.

## Data Availability

ARIC Study data is publicly available on the National Heart, Lung, and Blood Institute's Biologic Specimen and Data Repository Information Coordinating Center (NHLBI's BioLINCC).
