# Supplementary material for: Hospital Readmission After Traumatic Brain Injury Hospitalization in Community‐Dwelling Older Adults
Source: Ann Clin Transl Neurol. 2025 Nov 30;13(5):864–74. doi: 10.1002/acn3.70269 (PMC13161884; doi:10.1002/acn3.70269)
Supplement: Supplementary file 1 — Table S1: Table of discharge ICD‐9/10 codes for traumatic brain injury (TBI). Table S2: Table of discharge ICD‐9/10 codes for orthopedic injuries (including falls and fractures). Table S3: Standardized mean differences in variables comparing pre‐ versus post‐propensity score matching. [file ACN3-13-864-s001.docx]

**Online Only Supplemental Material**

Thomas R*, Law CA*, Casey JA, Mosley T, Gottesman RF, Diaz-Arrastia R, Elser H**, Schneider ALC**. Hospital Readmission after Traumatic Brain Injury Hospitalization in Community-Dwelling Older Adults.

*Co-first authors; these authors contributed equally.

**Co-senior authors; these authors contributed equally.

**Table of Contents**

Supplemental Table 1. Table of discharge ICD-9/10 codes for traumatic brain injury (TBI) ....................... 2

Supplemental Table 2. Table of discharge ICD-9/10 codes for orthopedic injuries (including falls and fractures) ....................................................................................................................................................... 3

Supplemental Table 3. Standardized mean differences in variables comparing pre- versus post-propensity score matching ............................................................................................................................................. 6

Supplemental Table 1. Table of discharge ICD-9/10 codes for traumatic brain injury (TBI).

| ***ICD-9*** | **Description** |
| --- | --- |
| 800.xx | Fracture of vault of skull |
| 801.xx | Fracture of base of skull |
| 803.xx | Other and unqualified skull fractures |
| 804.xx | Multiple fractures involving skull or face with other bones |
| 850.xx | Concussion |
| 851.xx | Cerebral laceration and contusion |
| 852.xx | Subarachnoid subdural and extradural hemorrhage following injury |
| 853.xx | Other and unspecified intracranial hemorrhage following injury |
| 854.xx | Intracranial injury of other and unspecified nature |
| 959.01 | Head injury, unspecified |
| ***ICD-10*** |  |
| S02.0 | Fracture of vault of skull |
| S02.1X | Fracture of base of skull |
| S02.8 | Fractures of other unspecified skull and facial bones |
| S02.91 | Unspecified fracture of skull |
| S04.02 | Injury of optic chiasm |
| S04.03X | Injury of optic tract and pathways |
| S04.04X | Injury of visual cortex |
| S06.X | Intracranial injuries, concussion, traumatic cerebral edema, diffuse and focal traumatic brain injury, traumatic epidural, subdural, and subarachnoid hemorrhage |
| S07.1 | Crushing injury of skull |

Supplemental Table 2. Table of discharge ICD-9/10 codes for orthopedic injuries (including falls and fractures).

| ***ICD-9*** | **Description** |
| --- | --- |
| **Falls** |  |
| E804.xx | Fall in on or from railway train |
| E833.xx | Fall on stairs or ladders while in water transport |
| E834.xx | Other fall from one level to another in water transport |
| E835.xx | Other and unspecified fall in water transport |
| E843.xx | Fall in, on, or from an aircraft, including any accident that occurs while boarding or exiting the aircraft |
| E880.0 | Accidental fall on or from stairs or steps |
| E880.1 | Accidental fall on or from sidewalk curb |
| E880.9 | Accidental fall on or from other stairs or steps |
| E881.0 | Accidental fall from a ladder |
| E881.1 | Accidental fall from a scaffolding |
| E882 | Accidental fall from or out of a building or other structure |
| E883.1 | Accidental fall into well |
| E883.2 | Accidental fall into storm drain or manhole |
| E883.9 | Accidental fall into a hole or other opening in a surface that is not a diving or jumping accident, a fall into a well, or a fall into a storm drain or manhole |
| E884.xx | Other accidental fall from one level to another |
| E885.xx | Accidental fall on the same level due to slipping, tripping, or stumbling |
| E886.xx | Accidental fall on the same level due to being pushed, shoved, or collided with by another person |
| E888.xx | Unspecified accidental fall |
| E913.3 | Accidental mechanical suffocation by falling earth or other substance |
| E917.xx | Striking against or struck with or without subsequent fall |
| E987.xx | Falling from man-made structures |
| V15.88 | History of fall |
| **Fractures** |  |
| 733.1x | Pathologic fracture |
| 733.93 | Stress fracture of the tibia or fibula |
| 733.94 | Stress fracture of the metatarsals |
| 733.95 | Stress fracture of other bone |
| 733.96 | Stress fracture of the femoral neck |
| 733.97 | Stress fracture of the shaft of the femur |
| 733.98 | Stress fracture of the pelvis |
| 800.xx | Fracture of vault of skull |
| 801.xx | Fracture of base of skull |
| 803.xx | Other and unqualified skull fractures |
| 804.xx | Multiple fractures involving skull or face with other bones |
| 805.xx | Fracture of vertebral column without mention of spinal cord injury |
| 806.xx | Fracture of vertebral column with spinal cord injury |
| 807.xx | Fracture of rib(s), sternum, larynx, and trachea |
| 808.xx | Fracture of the pelvis |
| 809.xx | Ill-defined fractures of bones of the trunk |
| 810.xx | Fracture of the clavicle |
| 811.xx | Fracture of the scapula |
| 812.xx | Fracture of the humerus |
| 813.xx | Fracture of the radius and ulna |
| 814.xx | Fracture of carpal bone(s) |
| 815.xx | Fracture of metacarpal bone(s) |
| 816.xx | Fracture of one or more phalanges of the hand |
| 817.xx | Multiple fractures of hand bones |
| 818.xx | Ill-defined fractures of upper limb |
| 819.xx | Multiple fractures involving both upper limbs and upper limb with rib(s) and sternum |
| 820.xx | Fracture of the neck of the femur |
| 821.xx | Fracture of other and unspecified parts of the femur |
| 822.xx | Fracture of the patella |
| 823.xx | Fracture of the tibia and fibula |
| 824.xx | Fracture of the ankle |
| 825.xx | Fracture of one or more tarsal and metatarsal bones |
| 826.xx | Fracture of one or more phalanges of the foot |
| 827.xx | Other, multiple, and ill-defined fractures of lower limb |
| 828.xx | Multiple fractures involving both lower limbs, lower with upper limb, and lower limb(s) with rib(s) and sternum |
| 829.xx | Fracture of unspecified bones |
| ***ICD-10*** |  |
| **Falls** |  |
| R29.6 | Repeated falls |
| v0011xx-v0089xx | Pedestrian conveyance accident with falls |
| w00xxxx-w19xxxx | Slipping, tripping, stumbling with falls |
| y30xxxx | Falling from high place |
| **Fractures** |  |
| M48.4 | Fatigue fracture of vertebra |
| M84.3 | Stress fracture that is not classified elsewhere |
| M96.A9 | Other fracture associated with chest compression and cardiopulmonary resuscitation |
| M96.62 | Fracture of humerus following insertion of orthopedic implant, joint prosthesis, or bone plate |
| M96.63 | Fracture of the radius or ulna that occurs after an orthopedic implant, joint prosthesis, or bone plate is inserted |
| M96.65 | Pelvic fracture that occurs after the insertion of an orthopedic implant, joint prosthesis, or bone plate |
| M96.66 | Fracture of femur following insertion of orthopedic implant, joint prosthesis, or bone plate |
| M96.67 | Fracture of tibia or fibula following insertion of orthopedic implant, joint prosthesis, or bone plate |
| M96.69 | Fracture of a bone other than the radius or ulna after an orthopedic implant, joint prosthesis, or bone plate is inserted |
| M97.X | Periprosthetic fracture around internal prosthetic joint |
| S02.0 | Fracture of vault of skull |
| S02.1X | Fracture of base of skull |
| S02.8 | Fractures of other unspecified skull and facial bones |
| S02.91 | Unspecified fracture of skull |
| S12X | Fracture of cervical vertebra and other parts of neck |
| S22X | Fracture of rib(s), sternum, and thoracic spine |
| S32X | Fracture of lumbar spine and pelvis |
| S42X | Fracture of shoulder and upper arm |
| S49.0xx | Physeal fracture of the upper end of humerus |
| S49.1xx | Physeal fracture of the lower end of humerus |
| S52X | Fracture of forearm |
| S59.0xxx | Physeal fracture of lower end of ulna |
| S59.1xxx | Physeal fracture of the upper end of radius |
| S59.2xxx | Physeal fracture of the lower end of radius |
| S62X | Fracture at wrist and hand level |
| S72X | Fracture of femur |
| S79.0xxx | Physeal fracture of upper end of femur |
| S79.1xxx | Physeal fracture of lower end of femur |
| S82X | Fracture of lower leg, including ankle |
| S89.0xxx | Physeal fracture of upper end of tibia |
| S89.1xxx | Physeal fracture of lower end of tibia |
| S89.2xxx | Physeal fracture of upper end of fibula |
| S89.3xxx | Physeal fracture of lower end of fibula |
| S92X | Fracture of foot and toe, except ankle |
| S99.1xxx | Physeal fracture of metatarsal |
| S99.2xxx | Physeal fracture of a toe phalanx |

Supplemental Table 3. Standardized mean differences in variables comparing pre- versus post-propensity score matching.

|  | TBI Versus Non-TBI Index Hospitalization | | TBI Versus Orthopedic Injury Index Hospitalization | |
| --- | --- | --- | --- | --- |
|  | Pre-Propensity Matching Standardized Mean Difference | Post-Propensity Matching Standardized Mean Difference | Pre-Propensity Matching Standardized Mean Difference | Post-Propensity Matching Standardized Mean Difference |
| Age at Index Hospitalization | -0.297 | 0.001 | -0.461 | -0.003 |
| Sex |  |  |  |  |
| Female | 0.712 | 0.00 | 0.465 | 0.000 |
| Male | -0.712 | 0.00 | -0.465 | 0.000 |
| Race/Center |  |  |  |  |
| Mississippi Black | -0.043 | 0.024 | 0.161 | 0.019 |
| North Carolina Black | 0.009 | -0.013 | 0.029 | 0.009 |
| Maryland White | -0.138 | 0.013 | -0.030 | -0.029 |
| Minnesota White | 0.161 | 0.000 | -0.035 | 0.005 |
| North Carolina White | 0.003 | -0.032 | -0.099 | 0.002 |
| Annual Household Income |  |  |  |  |
| <$35,000 | 0.024 | -0.044 | -0.116 | 0.014 |
| ≥$35,000 | -0.015 | 0.031 | 0.121 | -0.002 |
| Not Reported | -0.020 | 0.029 | -0.015 | -0.028 |
| Health Insurance | -0.007 | -0.027 | -0.087 | -0.044 |
| Marital Status |  |  |  |  |
| Divorced/Separated | 0.065 | -0.029 | 0.087 | 0.012 |
| Married | -0.067 | -0.017 | -0.140 | -0.011 |
| Never Married | 0.085 | 0.054 | 0.092 | -0.026 |
| Widowed | 0.045 | 0.000 | 0.051 | 0.057 |
| Unknown | -0.077 | 0.035 | 0.024 | 0.004 |
| Education |  |  |  |  |
| Less than High School | -0.176 | 0.046 | 0.007 | 0.006 |
| High School or Equivalent | 0.003 | -0.044 | -0.033 | -0.003 |
| Some College or Greater | 0.141 | 0.007 | 0.028 | -0.002 |
| Military Veteran | -0.387 | -0.009 | -0.292 | -0.012 |
| Physical Activity Index | -0.076 | -0.001 | -0.139 | 0.007 |
| Body Mass Index | -0.071 | 0.013 | -0.084 | 0.031 |
| Smoking |  |  |  |  |
| Current | -0.019 | -0.017 | 0.100 | 0.000 |
| Former | -0.172 | 0.021 | -0.164 | 0.014 |
| Never | 0.180 | -0.008 | 0.082 | -0.013 |
| Alcohol Consumption |  |  |  |  |
| Current | 0.081 | -0.004 | -0.044 | 0.015 |
| Former | -0.182 | -0.003 | -0.056 | 0.026 |
| Never | 0.084 | 0.007 | 0.103 | -0.042 |
| Hypertension | -0.180 | -0.012 | -0.092 | 0.019 |
| Hyperlipidemia | 0.008 | -0.027 | 0.006 | -0.007 |
| Diabetes | -0.182 | -0.004 | -0.010 | 0.010 |
| Coronary Heart Disease | -0.378 | -0.017 | -0.092 | 0.021 |
| Stroke | -0.190 | -0.003 | -0.069 | -0.07 |
| Days Between Study Enrollment and Index Hospitalization | -0.327 | 0.002 | -0.541 | 0.011 |

The following variables were ascertained at Visit 1 (1987-1989): sex, race/center, education, annual household income, health insurance status, marital status, military veteran status, physical activity index. The following variables were defined at the closest visit prior to the index hospitalization: body mass index, smoking, alcohol consumption, hypertension, hyperlipidemia, diabetes, coronary heart disease, stroke.
